# Non-Covalent Interaction between Polyubiquitin and GTP Cyclohydrolase 1 Dictates Its Degradation

**DOI:** 10.1371/journal.pone.0043306

**Published:** 2012-09-12

**Authors:** Yu Zhao, Huaiping Zhu, Ming-Hui Zou

**Affiliations:** 1 Section of Molecular Medicine, Department of Medicine, University of Oklahoma Health Science Center, Oklahoma City, Oklahoma, United States of America; 2 Department of Biochemistry and Molecular Biology, University of Oklahoma Health Science Center, Oklahoma City, Oklahoma, United States of America; Medical College of Wisconsin, United States of America

## Abstract

GTP cyclohydrolase 1 (GTPCH1) is the rate-limiting enzyme in the *de novo* synthesis of tetrahydrobiopterin (BH4). GTPCH1 protein degradation has been reported in animal models of several diseases, including diabetes mellitus and hypertension. However, the molecular mechanisms by which GTPCH1 is degraded remain uncharacterized. Here we report a novel non-covalent interaction between polyubiquitin and GTPCH1 *in vitro* and *in vivo*. The non-covalent binding of GTPCH1 to polyubiquitin via an ubiquitin-binding domain (UBD) results in ubiquitination and degradation. Ectopic expression of ubiquitin in cultured cells accelerated GTPCH1 degradation. In cultured cells and *in vitro* assays, Lys48-linked ubiquitin chains, but not Lys63-linked chains, interacted with GTPCH1 and targeted it for degradation. Consistently, proteasome inhibition attenuated GTPCH1 degradation. Finally, direct mutagenesis of an isoleucine (Ile131) in the hydrophobic patch of the GTPCH1 UBD affected its ubiquitin binding and the enzyme stability. Taken together, we conclude that GTPCH1 non-covalently interacts with polyubiquitin via an ubiquitin-binding domain. The polyubiquitin binding directs GTPCH1 ubiquitination and proteasome degradation.

## Introduction

GTP cyclohydrolase I (GTPCH1; EC 3.5.4.16) is the first, and rate-limiting, enzyme in tetrahydrobiopterin (BH4) biosynthesis that catalyzes the conversion of GTP to 7,8-dihydroneopterin 3′-triphosphate. BH4 is an essential cofactor required by aromatic amino acid hydroxylase and nitric oxide synthase (NOS) enzymes in the biosynthesis of the monoamine neurotransmitters serotonin, melatonin, dopamine, norepinephrine, epinephrine, and nitric oxide (NO), respectively [1]–[6]. Mutations in the GTPCH1 gene (GCH1) are associated with malignant phenylketonuria and hyperphenylalaninemia, as well as L-DOPA (Levodopa)-responsive dystonia [7]–[9]. Recent evidence indicates that GTPCH1 protein loss or inactivation, and the consequent BH4 deficiency, is widely found in cardiovascular and neurological disorders [8], [10]–[17]. However, the routes and mechanisms by which GTPCH1 is degraded remain undefined.

Ubiquitination typically occurs as a covalent modification of other proteins. Recently, non-covalent ubiquitination has also been described [18]–[22]. In non-covalent ubiquitination, ubiquitin signals are recognized and processed by ubiquitin-binding domains (UBDs) that form transient, non-covalent interactions either with ubiquitin or with ubiquitin chains. To date, more than 20 different families of UBDs had been identified [21]. Most UBDs fold into α-helical structures to contact ubiquitin or ubiquitin chains, such as ubiquitin-associated domain (UBA) and ubiquitin-interacting motif (UIM) [20], [23]. A hydrophobic patch in the UBD serves as a protein–protein interaction interface with a hydrophobic pocket on ubiquitin. In nearly all reported cases, Ile44 of ubiquitin contacts a conserved residue that is specific to each type of UBD [24]. The UBA domain is approximately 45 amino acids long and folds into a two- or three-helix bundle [18]. In the case of the UBA of the human DNA-repair protein, HHR23A, Leu355 of the hydrophobic patch mediates the binding with ubiquitin. Point mutants in the hydrophobic patch selectively affect ubiquitin binding [25].

UBDs interact with mono-ubiquitin and/or ubiquitin chains and possess multiple functions. The ubiquitin protein consists of 76 amino acids and has seven lysine residues (Lys6, Lys11, Lys27, Lys29, Lys33, Lys48, and Lys63), which allows for seven possible homotypic linkages and multiple heterotypic chains [26]. Different ubiquitin chains linked through the different lysine residues direct the marked proteins to different fates. In particular, the canonical Lys48 ubiquitin linkage is used to signal proteolysis [27]. Binding to a polyubiquitin chain (of at least four ubiquitins) is necessary for targeting proteins for degradation [28].

GTPCH1 degradation is proteasome dependent and ubiquitination might be involved in this process [13]. However, the molecular mechanism by which the ubiquitin-proteasome system targets GTPCH1 is unknown. The present study extends our previous reports by investigating the molecular mechanisms of GTPCH1 ubiquitination and its consequent degradation. Our results show that non-covalent interaction between ubiquitin and GTPCH1 via a UBD dictates its degradation.

## Materials and Methods

### Materials

Antibodies against GTPCH1, FLAG tag, HA tag and anti-FLAG resin were obtained from Sigma-Aldrich (St. Louis, MO). Antibodies against ubiquitin, glutathione S-transferase (GST), and protein A/G plus agarose were obtained from Santa Cruz Biotechnology Inc. (Santa Cruz, CA). The proteasome inhibitors MG132 and lactacystin were purchased from BioMol (Plymouth Meeting, PA). Tetraubiquitin (Ub4), mixed ubiquitin chains (Ub2∼7), UIM-agarose for the enrichment of ubiquitin, and sample buffer were obtained from Boston Biochem Inc. (Cambridge, MA). Glutathione (GSH) beads for recombinant protein purification were from Amersham Pharmacia Biotech (Uppsala, Sweden). Full-length human GCH1 cDNA and lipofectamine 2000 were obtained from Invitrogen (Carlsbad, CA) and QuikChange site-directed mutagenesis kits were purchased from Stratagene (La Jolla, CA). Restriction enzymes *EcoR*I and *Sal*I were from New England Biolabs (Ipswich, MA). Qiaquick DNA isolation kits were from Qiagen (Valencia, CA). Primers were designed and purchased from Sigma-Aldrich (St. Louis, MO). All other chemicals were purchased from Sigma-Aldrich (St. Louis, MO) unless otherwise indicated.

### Animals

C57BL/6J mice (8–12 weeks of age) were obtained from the Jackson Laboratory (Bar Harbor, ME). Mice were housed in temperature-controlled cages with a 12-hour light/dark cycle and given free access to water and food. The animal protocol was reviewed and approved by the Institutional Animal Care and Use Committee at the University of Oklahoma Health Sciences Center.

### Cell culture

HEK293 cells (Invitrogen, Carlsbad, CA) were grown in Dulbecco's modified Eagle's medium supplemented with 1% (v/v) penicillin-streptomycin and 10% (v/v) fetal calf serum. Mouse endothelial cells SVEC4-10 (ATCC, Manassas, VA) were grown in endothelial basal media supplemented with 2% fetal bovine serum, penicillin (100 units/ml), and streptomycin (100 µg/ml). Cultured cells were used between passages 4 and 10. All cells were incubated at 37°C in a humidified atmosphere of 5% CO2.

### Plasmids and mutagenesis

Full-length GCH1 cDNA was synthesized by PCR amplification of the full-length human GCH1 cDNA. Amplification was carried out for 35 cycles (95°C for 30 s, 55°C for 30 s, and 72°C for 60 s) using a Bio-Rad Thermal Cycler System (Bio-Rad). The primers used in the amplification incorporated *EcoR*I and *Sal*I restriction sites for directional cloning of the amplified DNA into expression vectors. The amplified DNA with a C-terminal FLAG tag was digested with *EcoR*I and *Sal*I restriction enzymes and directionally cloned into the pCI-neo plasmid (Promega) for mammalian protein expression. Mutants of FLAG-GCH1 (I131A or I131L) were made using the Stratagene QuikChange site-directed mutagenesis kit, following the manufacturer's protocol. GST-GTPCH1 (42–250) was amplified by PCR and inserted into the pGEX-4T-2 vector (GE Healthcare, formerly Amersham Biosciences) for bacterial protein expression. N-terminal truncations of GST-GTPCH1 NΔ1 (30–85), NΔ2 (95–142), and NΔ3 (128–250) were PCR amplified. The following plasmids were obtained from Addgene (www.addgene.org): pRK5-HA-Ubiquitin-WT (HA-Ub-WT, Addgene ID 17608), pRK5-HA-Ubiquitin-K48 (K48-only-Ub, Addgene ID 17605) and pRK5-HA-Ubiquitin-K63 (K63-only-Ub, Addgene ID 17606). All constructed plasmids were fully sequenced before use. Details of plasmids construction are available upon request.

### Expression and purification of recombinant GTPCH1

GST-GTPCH1 (42–250) plasmids and truncations were expressed in the BL21 (DE3) *E. coli* strain using standard techniques. Transformants were used to inoculate 3 mL of Luria Bertani (LB) media and the cultures were incubated overnight at 37°C with agitation at 220 rpm. These cultures were used to inoculate 1 L of LB media and incubated at 37°C with agitation at 220 rpm until the optical density at 600 nm reached 0.6. Isopropyl-β-d-thiogalacto-pyranoside was added to a final concentration of 0.7 mM and the culture was incubated for an additional 5 hours. Cells were resuspended in lysis buffer [100 mM Tris-HCl pH 8.5, 100 mM NaCl, 10% glycerol, 1% Triton X-100, and protease inhibitors (Calbiochem)] and lysed by brief sonication. The GST fusion proteins were purified using GSH beads in accordance with the manufacturer's protocol. Protein purity was demonstrated by 12% sodium dodecyl sulfate polyacrylamide gel electrophoresis (SDS-PAGE) and the concentration of the purified protein was determined by the BCA Protein Assay (Thermo Scientific Pierce).

### *In Vitro* binding assays

Tetraubiquitin chains (Boston Biochem) were used in binding assays [29] with recombinant GST-GTPCH1 or GTPCH1 mutants immobilized on GSH beads. Briefly, GST-GTPCH1 or mutants on GSH beads were incubated in binding buffer (100 mM Tris-HCl pH 8.5, 100 mM NaCl, 10% glycerol, 1% Triton) with a molar excess of chain ligand overnight at 4°C. After washing with binding buffer five times, bound ubiquitin chains were eluted with sample buffer and resolved by SDS-PAGE, and Western blotting was performed with an anti-ubiquitin antibody at a dilution of 1∶1000 followed by ECL (Thermo Scientific Pierce) to detect the ubiquitin chains.

### Polyubiquitin chains overexpression and GST pull-down assay

Cell extracts were prepared with the accumulation of ubiquitin chains as described [27] with minor modifications. Briefly, hemagglutinin-tagged ubiquitin (HA-Ub) chains were produced by transfecting HEK293 cells with HA-Ub plasmids (Addgene ID 17608) for 3 days. Lysates from cells with the overexpressed HA-Ub chains were incubated overnight at 4°C with purified GST fusion proteins on GSH beads. Beads were then washed four times with lysis buffer. Bound proteins were eluted and boiled with reduced sample buffer (Boston Biochem), resolved by SDS-PAGE, and immunoblotted with the indicated antibodies.

### Immunoprecipitation and western blots

HEK293 cells were transfected with FLAG-tagged GTPCH1 or GTPCH1 mutants using Lipofectamine 2000 (Invitrogen) for 2 or 3 days. Cells were harvested and resuspended in lysis buffer and immunoprecipitated with the polyclonal ubiquitin antibody or the FLAG resin antibody. Protein was measured using the BCA protein assay reagent (Pierce, USA). Immunoblotting was carried out using standard techniques with the FLAG, GTPCH1, and ubiquitin antibodies. Protein bands were visualized using the ECL reagent (Thermo Scientific Pierce). Protein band densities were analyzed using the spot density analysis software: Quantity one (Bio-Rad).

### Ubiquitination assays

Ubiquitination assays were performed by immunoprecipitation. Briefly, HEK293 cells were transfected with the indicated plasmids and disrupted in lysis buffer, followed by immunoprecipitation and immunoblotting with the indicated antibodies.

### Statistical analysis

Results are expressed as mean±SD. Statistical significance for comparisons between 2 groups was calculated using the 2-tailed student t test. To assess comparisons between multiple groups, ANOVA followed by the Bonferroni procedure was performed using the Graph-Pad Prism 4 program (GraphPad Software, Inc, San Diego, CA). A probability value of <0.05 was considered to be statistically significant.

## Results

### GTPCH1 interacts with polyubiquitin in mouse heart and lung *in vivo*

**Figure 1:**
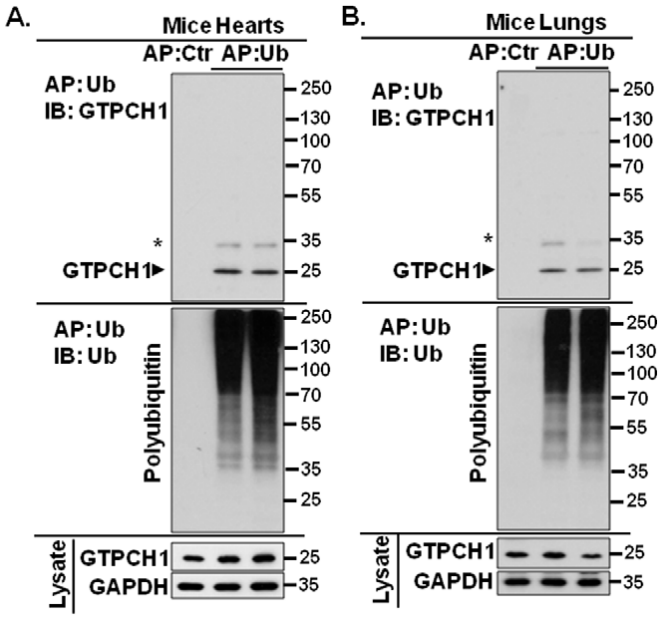
GTPCH1 non-covalently interacts with polyubiquitin in mouse heart and lung *in vivo*. Mice tissues (n = 4) were isolated and the homogenates were generated as described in Experimental Procedures. The tissue lysates were incubated with UIM-agarose for ubiquitin affinity precipitation (AP). The proteins that interacted with polyubiquitin chains were analyzed by western blot using specific antibodies. **A.** GTPCH1 and polyubiquitin chains interaction in mouse heart and **B.** mouse lung. Control beads without UIM were used as a negative control are included in all experiments. The black triangle (▸) indicates the bound GTPCH1 by polyubiquitin chains and the asterisk (*) indicates a non-specific band. Results were obtained from four mice.

Protein ubiquitination is a process by which ubiquitin is covalently attached to the ε-amino group of lysine residues in a substrate protein. In addition, ubiquitin also non-covalently interacts with target proteins through the UBD [21]. To determine if GTPCH1 interacts with ubiquitin *in vivo*, we used ubiquitin affinity precipitation (AP) to enrich ubiquitin-positive proteins in the lysates of mouse heart and lung. The proteins that interact with polyubiquitin were analyzed by denatured SDS-PAGE and western blot. As shown in Fig. 1, both heart and lung express GTPCH1, and ubiquitin AP yielded typically strong, diffuse bands of polyubiquitin chains in heart and lung (Fig. 1).

The co-precipitated GTPCH1 with polyubiquitin was determined by western blotting of the ubiquitin-enriched preparation with the GTPCH1 antibody showed a clear band with a molecular weight of 25 kDa (upper panel, Fig. 1), which is same as the molecular weight of GTPCH1 in the lysates of heart and lung. In addition, we also detected a band at 33 kDa in the both the heart and lung fractions (Fig. 1). Because we did not observe a band in the immunoblots with the ubiquitin antibody in the ubiquitin enrichment (Fig. 1), the 33-kDa band is likely nonspecific. Taken together, our data indicate that GTPCH1 interacts with polyubiquitin in mouse heart and lung *in vivo*. Because the co-precipitated GTPCH1 by polyubiquitin was identified only at 25 kDa, this result suggests that polyubiquitin non-covalently binds GTPCH1 (Fig. 1).

### GTPCH1 binds polyubiquitin in mouse endothelial cells

**Figure 2:**
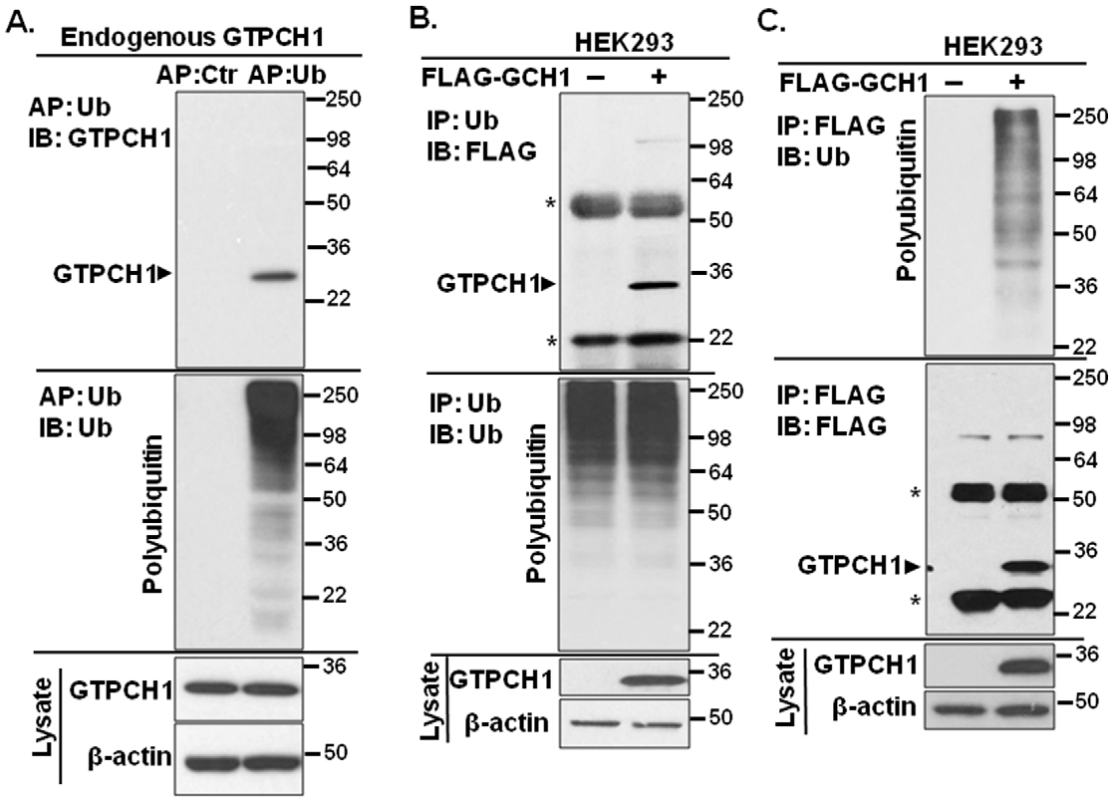
GTPCH1 binds polyubiquitin in cultured cells. **A.** Endogenous GTPCH1 binds polyubiquitin in mouse endothelial cells (SVEC4-10). Mouse endothelial cells were grown to confluence and cell lysates were incubated with UIM-agarose for ubiquitin affinity precipitation (AP). The interaction of GTPCH1 with polyubiquitin chains was analyzed by immunoblotting. The black triangle (▸) indicates the bound GTPCH1 by polyubiquitin chains. Control beads without UIM were used as a negative control. **B.** Co-immunoprecipitation of GTPCH1 with polyubiquitin in GTPCH1 overexpressed HEK293 cells. FLAG-GCH1 plasmids or empty vectors were transfected into HEK293 cells for 2 days. Cell lysates were incubated with the ubiquitin antibody and immunoprecipitated using protein A/G beads. The asterisk (*) indicates IgG bands. HEK293 transfected with empty vectors were used as a negative control. **C.** Polyubiquitin chains were co-immunoprecipitated with GTPCH1. HEK293 cell lysates expressing FLAG-GTPCH1 or control vector were incubated with anti-FLAG resin. Proteins that co-immunoprecipitated with FLAG-GTPCH1 were analyzed by immunoblotting using the indicated antibodies. The blot shown is representative of five independent experiments.

GTPCH1 is constitutively expressed in endothelial cells and its degradation and the consequent BH4 deficiency are responsible for endothelial dysfunction [11]–[13]. Because GTPCH1 interacts with polyubiquitin in mouse heart and lung (Fig. 1), we questioned whether GTPCH1/Ub interaction occurs in cultured cells. As shown in Fig. 2A, ubiquitin AP enrichment but not the AP control resulted in intense but diffuse polyubiquitin signals in mouse endothelial cells (SVEC4-10). The co-precipitated GTPCH1 by polyubiquitin was determined by western blotting with the GTPCH1 antibody (Fig. 2A). Importantly, only one major band (∼25 kDa) was detected for the co-precipitated GTPCH1 by polyubiquitin, suggesting a direct non-covalent interaction between polyubiquitin and GTPCH1 in endothelial cells.

### GTPCH1 is ubiquitinated in HEK293 cells ectopically expressing GTPCH1

To further determine if GTPCH1 and polyubiquitin directly interact in intact cells, the FLAG tagged-GCH1 (FLAG-GCH1) plasmid was transfected into HEK293 cells. Two days post-transfection, cell lysates were first immunoprecipitated using the ubiquitin antibody. As expected, GTPCH1 co-immunoprecipitated with polyubiquitin when blotted using the FLAG-specific antibody (Fig. 2B). Notably, there was no shift in molecular weight (Fig. 2B).

It is important to confirm the GTPCH1/Ub interaction using “reverse” immunoprecipitation (IP). HEK293 cells expressing FLAG-GTPCH1 were first immunoprecipitated using the FLAG antibody. The western blot shows a typical smeared bands when probed with the ubiquitin antibody (Fig. 2C), suggesting that GTPCH1 interacts with polyubiquitin in the lysates. However, the same blots show one major band at 30 kDa when blotted with the FLAG antibody to detect GTPCH1 (Fig. 2C). Taken together, these results indicate co-immunoprecipitation (co-IP) of GTPCH1 with polyubiquitin chains.

### Ubiquitin overexpression decreases GTPCH1 levels

**Figure 3:**
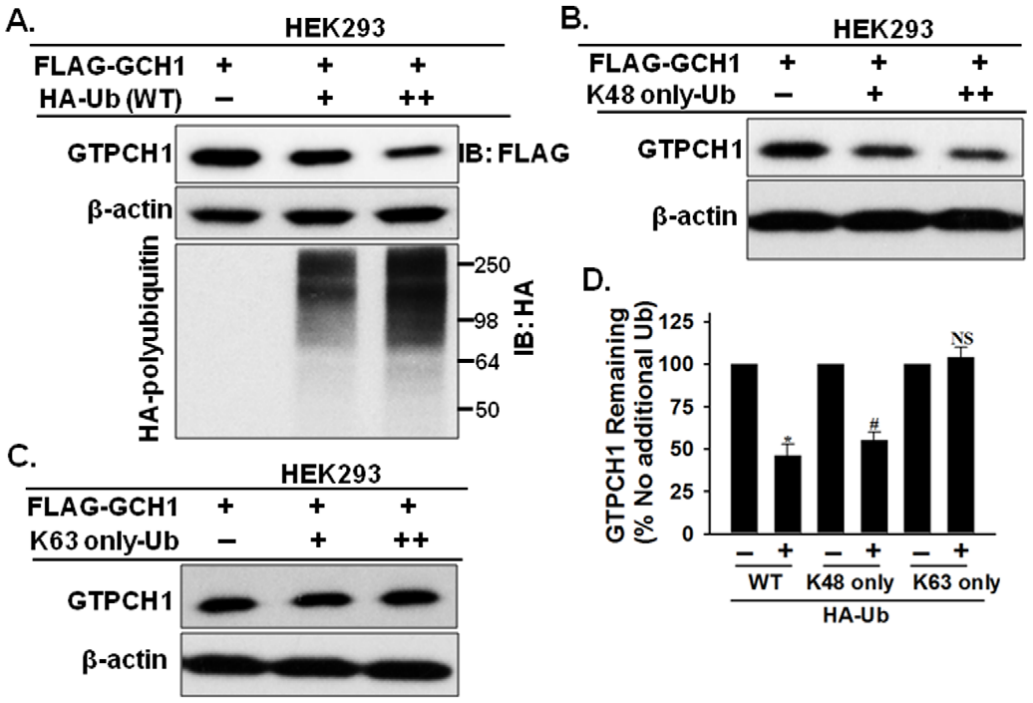
Interaction of GTPCH1 with Lys48 linked polyubiquitin chains promotes its degradation. **A.** HEK293 cells were co-transfected with FLAG-GCH1 and HA tagged ubiquitin plasmids (HA-Ub) for 2 days, cells were lysed, and the proteins were analyzed by immunoblotting using the indicated antibodies. The double plus symbol (**++**) indicates two folds of HA-Ub plasmids. HEK293 cells were co-transfected with FLAG-GCH1 plasmids and **B.** Lys48-only ubiquitin plasmids (K48 only-Ub) or, **C.** Lys63-only ubiquitin plasmids (K63 only-Ub). **D.** Compare of the GTPCH1 level with or without ubiquitin plasmids overexpression. The blot shown is representative of three independent experiments. Data are shown as mean±standard deviation (n = 3). *^#^ *P*<0.05 vs. control (without ubiquitin plasmids overexpression). NS indicates *P*>0.05 vs. control.

Increasing the amount of available ubiquitin as well as fluctuations in the intracellular ubiquitin content can actively alter the degradation of target proteins [30]. Because GTPCH1 interacts with polyubiquitin in heart and lung (Fig. 1), as well as in endothelial cells (Fig. 2), it was important to determine the role of polyubiquitin on GTPCH1 regulation. To this end, we co-expressed varying amounts of the HA-Ub plasmids (HA-Ub-WT, Addgene ID 17608) with the same amount of FLAG-GCH1 plasmids in HEK293 cells for 2 days. As expected, overexpression of HA-Ub plasmids markedly increased the amount of HA-tagged polyubiquitin chains, which are typical smeared bands in the western blot when probed with the HA antibody (bottom panel in Fig. 3A). Concomitantly, increased expression of HA-Ub significantly decreased the level of FLAG-tagged GTPCH1 proteins (Fig. 3A).

### Lys48-linked ubiquitin chains target GTPCH1 for degradation

Ubiquitin has seven lysine residues, including K48 and K63, which serve as points for ubiquitination. Ubiquitin chains linked through different lysine residues direct the fates of the marked proteins. For example, the canonical Lys48-linked ubiquitin chain (K48-Ub) is used to signal proteolysis, and the Lys63 linkage (K63-Ub) is used in several nonproteolytic signaling pathways [27]. Because overexpression of ubiquitin decreased GTPCH1 expression, we next determined which type of ubiquitin chain dictates GTPCH1 degradation. To this end, we co-expressed in HEK293 cells increasing amounts of plasmids expressing K48-only-Ub (K48 only, other lysines mutated to arginines, Addgene ID 17605) or K63-only-Ub (K63 only, other lysines mutated to arginines, Addgene ID 17606) with a fixed amount of FLAG-GCH1 plasmids. As depicted in Fig. 3B, GTPCH1 degradation is associated with increasing amounts of K48-only-Ub. In contrast, Lys63-linked ubiquitin chains (K63-only-Ub) had no effect on GTPCH1 degradation (Fig. 3C). Taken together, our results suggest that GTPCH1 interacts with Lys48-linked ubiquitin chains and that this controls GTPCH1 degradation (Fig. 3D).

### Inhibition of the 26S proteasome causes GTPCH1/Ub complex accumulation

**Figure 4:**
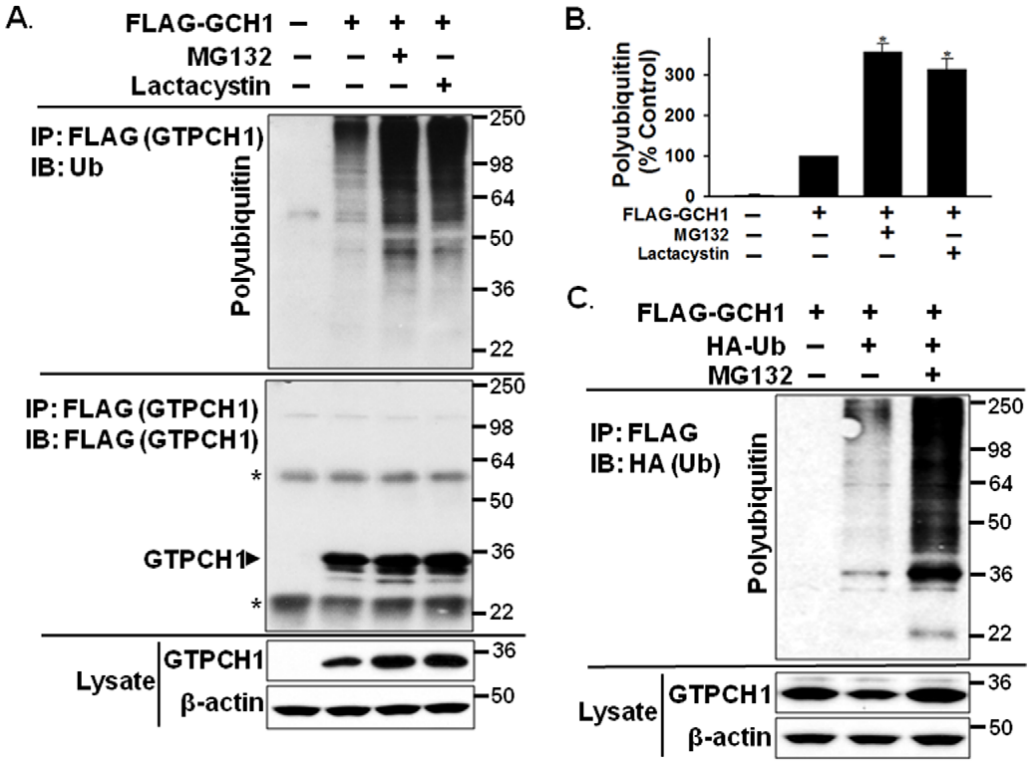
Inhibition of the 26S proteasome causes the accumulation of the GTPCH1/Ub complex. **A and B.** HEK293 cells were transfected with FLAG-GCH1 or empty vector (control) for 2 days and treated with the 26S proteasome inhibitors MG132 or lactacystin (Lac, irreversible inhibitor) for 6 hours. Lysates from cells expressing FLAG-GTPCH1 were immunoprecipitated with anti-FLAG resin and immunoblotted using the indicated antibodies. HEK293 transfected with empty vectors were used as a negative control. The asterisk (*) indicates IgG bands. **C.** HEK293 cells were co-transfected with FLAG-GCH1 and HA-Ub plasmids and treated with or without MG132 for 6 hours. Lysates from cells expressing FLAG-GTPCH1 were immunoprecipitated with anti-FLAG resin and immunoblotted with the FLAG, HA, or β-actin antibodies. The blot shown is representative of three independent experiments.

We next determined whether the degradation of ubiquitinated GTPCH1 requires the proteasome. To this end, we inhibited the proteasome in HEK293 cells overexpressing FLAG-GTPCH1 using MG132 (a reversible inhibitor of proteasome) or lactacystin (Lac, irreversible inhibitor). Consistent with the data presented in Fig. 2C, ubiquitin chains co-immunoprecipitated with FLAG-GTPCH1 (Fig. 4A, Lanes 1 and 2). Importantly, proteasome inhibition using both MG132 and lactacystin dramatically increased the amount of ubiquitin chains bound to FLAG-GTPCH1 and also caused the accumulation of the GTPCH1/Ub complex (∼3-fold, Fig. 4A and B).

To avoid the possible nonspecific binding due to the overexpression of GTPCH1, we further confirmed the accumulation of the GTPCH1/Ub complex by using co-transfection of HA-Ub plasmids (Addgene ID 17608) with FLAG-GCH1 plasmids in HEK293 cells. The cell lysates overexpressed HA tagged ubiquitin chains and FLAG-GTPCH1 proteins were used for co-immunoprecipitation (co-IP) assay by anti-FLAG resin (Sigma). As shown in Fig. 4C, the co-precipitated HA tagged ubiquitin chains with GTPCH1 was determined by western blotting as the typical smear signal (Fig. 4C, Lanes 2), while the cells transfected with FLAG-GCH1 only (as a negative control) have no staining for the co-IP assay and blotting with anti-HA antibody (Fig. 4C, Lanes 1). Importantly, inhibition of the proteasome led to the accumulation of the GTPCH1/HA-Ub complex (Fig. 4C, Lanes 3). Together, these data suggest that GTPCH1 specifically binds ubiquitin, and that the GTPCH1/Ub complex is subsequently degraded by the proteasome.

### GTPCH1 non-covalently binds synthesized tetraubiquitin *in vitro*

**Figure 5:**
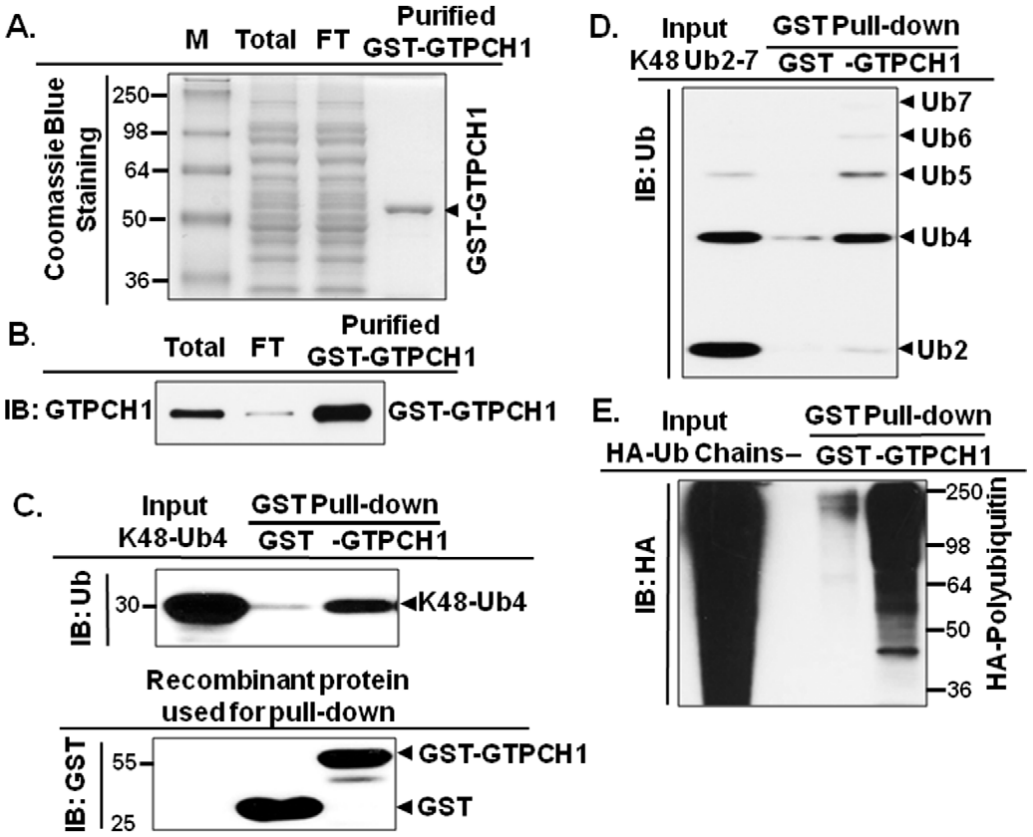
GTPCH1 non-covalently interacts with polyubiquitin chains *in vitro*. **A**. Generation of recombinant GTPCH1 for pull-down assays. As described in Experimental Procedures, N-terminal GST-fused GTPCH1 (42–250) was expressed in *E. coli* (BL21), purified by GSH beads, and analyzed by SDS-PAGE stained with Coomassie blue. M, molecular marker; Total, *E. coli* lysates expressing GST-GTPCH1 (42–250); FT: Flow Through, lysates after purification by GSH beads column. **B**. Identification of purified GST-GTPCH1 (42–250) by immunoblotting with GTPCH1 antibody. **C.** Recombinant GST-GTPCH1 binds synthesized tetraubiquitin chains, or **D.** mixed ubiquitin chains (Ub2∼7 chains). **E.** GST pull-down assays of recombinant GST-GTPCH1 with HA tagged ubiquitin chains expressed in HEK293 cell. The interacting proteins with GST-GTPCH1 *in vitro* were analyzed by immunoblotting with the HA antibody. Data for GST alone (as a negative control) is included in all experiments. **F.** FLAG-GTPCH1 pull-down assay. As described in Experimental Procedures, recombinant FLAG-GTPCH1 bound to anti-FLAG resin was incubated with tetraubiquitin chains (Ub4). Bound proteins were detected by immunoblotting. The anti-FLAG resin only was incubated with Ub4 as a negative control. The blot shown is representative of three independent experiments.

We further verified the GTPCH1/Ub interaction *in vitro*. The recombinant GST-tagged GTPCH1 (GST-GTPCH1) proteins used in this study were generated as described in Experimental Procedures. The purity of GST-GTPCH1, which appears as a single band in Coomassie Blue staining (Fig. 5A), was further confirmed in western blot experiments (Fig. 5B).

Because K48-linked, but not K63-linked, ubiquitin chains dictated GTPCH1 degradation in cultured cells (Fig. 3), we further used synthesized Lys48-linked tetraubiquitin (K48-Ub4, Boston Biochem) for *in vitro* assay. As shown in Fig. 5C, recombinant GST-GTPCH1, but not GST only control, interacted with K48-Ub4. Consistently, GST-GTPCH1 specifically interacted with Ub2∼7 chains (Fig. 5D).

### GTPCH1 binds overexpressed ubiquitin chains *in vitro*

We next investigated the interaction properties of GST-GTPCH1 with overexpressed ubiquitin chains from cell lysates. HA-Ub chains were produced by transfecting HEK293 cells with HA-Ub plasmids and the lysates from cells expressing HA-Ub were used in GST pull-down assays with purified GST-GTPCH1. As shown in Fig. 5E, GST-GTPCH1 directly interacted with the HA-Ub chains.

### Identification of an ubiquitin-binding domain in GTPCH1

Next, we determined which domains of GTPCH1 participate in ubiquitin binding. Ubiquitin signals are recognized and processed by UBDs that form transient, noncovalent interactions with ubiquitin or the ubiquitin chains [21]. Most UBDs fold into α-helical structures to contact ubiquitin or ubiquitin chains [20], [23], and the conserved hydrophobic core residues are the key features for some types of UBDs, such as ubiquitin-associated (UBA) domain and CUE domain (coupling of ubiquitin conjugation to endoplasmic reticulum degradation domain) [21], [25], [31], [32]. “GFP-loop” [32] and putative protein-protein interaction site [25], [31], which including the GFP-loop is for protein-protein interactions, as well as ubiquitin binding, are the most impotent features for ubiquitin-associated (UBA) domain [25].

**Figure 6:**
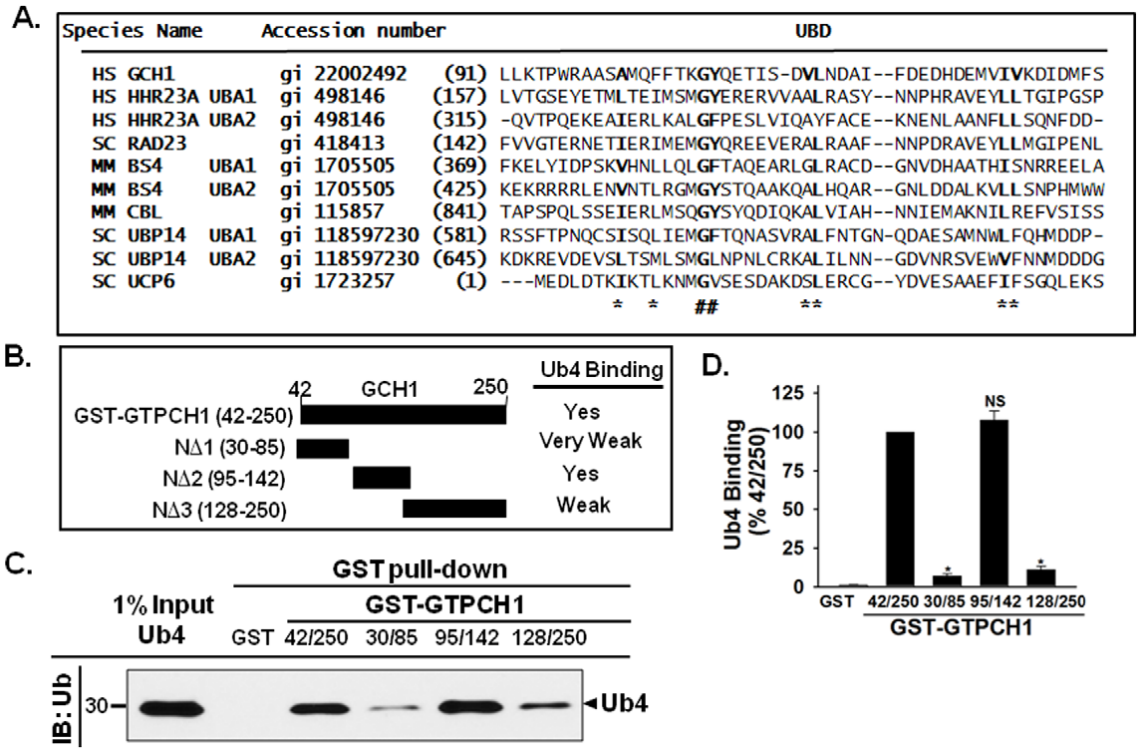
The ubiquitin-binding domain (UBD) of GTPCH1 mediates the interaction with ubiquitin chains. **A**. Alignment of ubiquitin binding domains found in multiple proteins. The accession numbers are for GenBank (gi) and residue numbers of the sequences are shown on the left. Below, (*) indicates conserved hydrophobic core residues and (#) indicates conserved “GFP-loop”. Abbreviations used: HS, human; SC, yeast; MM, mouse. **B**. Schematic of the GTPCH1 deletion mutants. **C & D**. GST-tagged GTPCH1 (42–250) or deletion mutants (NΔ1∼NΔ3) were expressed in *E. coli*. Purified GST-GTPCH1 proteins bound to GSH beads were incubated with the synthesized tetraubiquitin chains (Ub4). The bound proteins were detected by immunoblot using the ubiquitin antibody. GST alone was used as a negative control. The blot shown is representative of three independent experiments. Data are shown as mean±standard deviation (n = 3). *P<0.05 vs. control. NS indicates *P*>0.05 vs. control.

Sequence alignment indicated that GTPCH1 contains a UBD (Fig. 6A). We found almost all the conserved hydrophobic residues (indicated as * and #, Fig. 6A), which is important to form the hydrophobic core for ubiquitin binding. In GTPCH1, the residues Gly 108 and Tyr 109 are highly conserved and have the potential to form “GFP-loop” in GTPCH1 (indicated as #, Fig. 6A). There is also a conserved hydrophobic residue Ile 131 of GTPCH1, corresponding to Leu 355 of HHR23A (indicated as *, Fig. 6A).

Due to the sequence alignment, we cloned three GTPCH1 deletion mutants (NΔ1∼3, Fig. 6B) as GST fusion proteins and expressed each mutant in *E. coli* (BL21). After purification, we surveyed their interaction properties using pull-down assays with tetraubiquitin (Ub4). As depicted in Fig. 6C and 6D, both GTPCH1 N-terminal truncation mutants NΔ1 (30–85) and NΔ3 (128–250) very weakly interacted with Ub4. In contrast, the GTPCH1 truncation NΔ2 (95–142) binds Ub4 very well as compared to GST GTPCH1 (42–250). Together, these data suggest that residues 95–142 comprise the GTPCH1 UBD and mediate the interaction between GTPCH1 and ubiquitin.

### Mutants of GTPCH1 isoleucine 131 in the hydrophobic patch affect its ubiquitination and stability

Hydrophobic patches serve as protein-protein interaction interfaces between the UBD and ubiquitin. UBD point mutants in the hydrophobic patch selectively affect ubiquitin binding. For example, in the second UBA (UBA2) of the human DNA-repair protein, HHR23A, the residues Gly 331, Phe 332 and Pro 333 form a sharp turn (which is GFP-loop) with Gly 331 and Phe 332 being highly conserved. GFP-loop connects with conserved Leu 355 and Leu 356 and mediated the ubiquitin binding. Mutation of Leu 355 disrupted HHR23A and ubiquitin binding [25].

**Figure 7:**
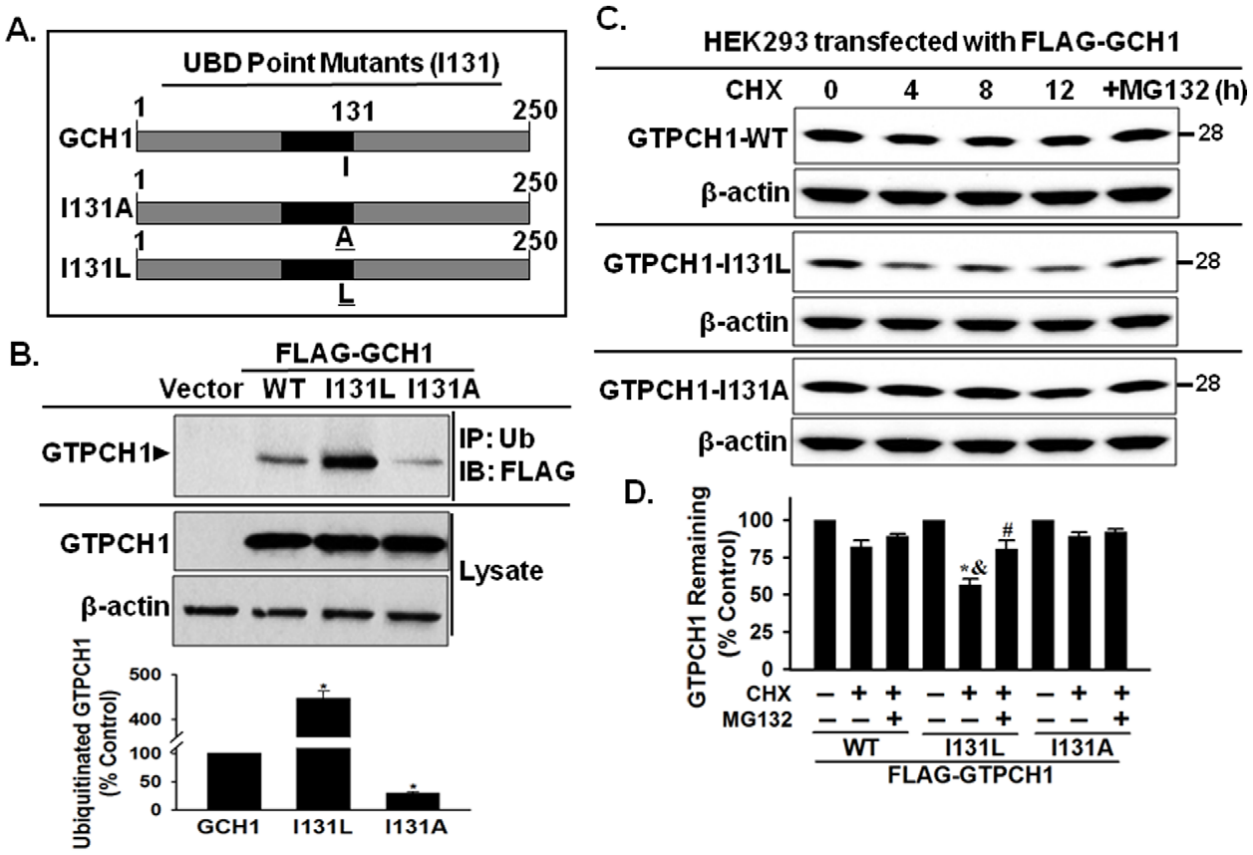
Mutants of GTPCH1 isoleucine 131 in the hydrophobic core of UBD affect polyubiquitin binding. **A**. Diagram of GTPCH1 point mutants in the hydrophobic core. **B**. GTPCH1 mutants affect ubiquitin binding. HEK293 cells were transfected with plasmids FLAG-GCH1 or mutants (I131A or I131L). Lysates from cells expressing FLAG-GTPCH1 and mutants were incubated with the ubiquitin antibody and immunoprecipitated using protein A/G beads. The proteins that co-immunoprecipitated with polyubiquitin were analyzed by immunoblot using specific antibodies. HEK293 transfected with empty vectors were used as a negative control. **C & D.** GTPCH1 isoleucine 131 affects protein stability. FLAG-tagged GTPCH1 (GTPCH1-WT) or I131 mutants (I131L or I131A) were transfected into HEK293 cells. After the transfection for 2 days, the cells were treated with CHX (100 µg/mL). At the times indicated, cells were harvested to assay GTPCH1 levels. The blot shown is representative of three independent experiments. Data are shown as mean±standard deviation (n = 3). *P<0.05 vs. control (without CHX treating). ^&^ *P*<0.05 vs. GTPCH1-WT plus CHX (without MG132). ^#^ *P*<0.05 vs. I131L plus CHX.

Sequence alignment of GTPCH1 with several representative UBD domains indicates that I131 in GTPCH1 is a highly conserved hydrophobic amino acid in the hydrophobic patch, which is same as the conserved L355 (Leu 355) in the second UBA (UBA2) of HHR23A (Fig. 6A). To determine whether I131 in the potential hydrophobic patch of GTPCH1 affects its ubiquitination, we constructed several point mutants of I131 of GTPCH1 (Fig. 7A) and transfected into HEK293 cells for 2 days. Co-immunoprecipitation (co-IP) was used to compare the interaction of GTPCH1 with ubiquitin. As expected, GTPCH1 co-immunoprecipitated with ubiquitin when blotted using the FLAG-specific antibody (lane 2, Fig. 7B). Notably, a point mutation that changes isoleucine (I131) into alanine (A) abolished the interaction with ubiquitin (Fig. 7B). Interestingly, a GTPCH1 point mutation of the isoleucine residue to leucine (I131L) dramatically increases ubiquitin binding (approximately 4.5-fold, Fig. 7B).

Next, we determined whether the point mutation of I131 also affects the stability of GTPCH1. Plasmids encoding FLAG-tagged WT GTPCH1 (FLAG-GCH1) or I131 mutants (I131L or I131A) were transfected into HEK293 cells. After the transfection for 2 days, the cells were treated with CHX (100 µg/mL). At the times indicated, cells were harvested to assay GTPCH1 levels. As shown in Fig. 7C and 7D, the stability of I131L GTPCH1 was dramatically decreased, compared with WT GTPCH1. Importantly, the accelerated degradation of I131L can be reversed by proteasome inhibitor MG132 (Fig. 7C). Taken together, these data suggest that I131 is the core residue of the hydrophobic patch and the UBD mediates GTPCH1 ubiquitin binding and the subsequent degradation.

## Discussion

In this study, we have identified a novel non-covalent interaction between polyubiquitin and GTP cyclohydrolase 1 *in vitro* and *in vivo*. Moreover, the binding of GTPCH1 with polyubiquitin chains via an ubiquitin-binding domain (residues 95–142) directs GTPCH1 ubiquitination and proteasome degradation. Mutation of the conserved Ile131 in the hydrophobic patch of the GTPCH1 UBD selectively affects its ubiquitin binding. Lys48-linked, but not Lys63-linked polyubiquitin chains bound and directed 26S proteasome-dependent GTPCH1 degradation.

The major finding of this study is that GTPCH1 non-covalently binds polyubiquitin chains *in vitro* and *in vivo*. Ubiquitin is a 76-amino acid protein and typically covalently modifies other proteins via polyubiquitin chains [33]. Target proteins that are covalently bound to varying chain lengths of ubiquitin cause corresponding molecular weight shifts in denatured SDS-PAGE. However, more recently, roles for non-covalent ubiquitination have been proposed [18]–[22]. In non-covalent ubiquitination, target proteins form transient, non-covalent interactions with ubiquitin. In the present study, there are several lines of evidence that are consistent with the hypothesis that the non-covalent interaction between polyubiquitin and GTPCH1 mediates its ubiquitination. First, GTPCH1 binds ubiquitin chains but there was no “classical” molecular weight shift of ubiquitinated-GTPCH1 (Ub-GTPCH1) in denatured SDS-PAGE from HEK293 cells expressing GTPCH1 (Fig. 2B and 2C), cultured endothelial cells (Fig. 2A), or mouse heart and lung *in vivo* (Fig. 1). Second, recombinant GTPCH1 directly interacts with polyubiquitin chains *in vitro* (Fig. 5). Finally, the GTPCH1 linkage specifically and preferentially bound high-molecular-weight ubiquitin chains (Fig. 5D and 5E), which is a typical ubiquitin binding property [34], [35]. Taken together, our results strongly indicate that there is a non-covalent interaction between GTPCH1 and polyubiquitin.

The present study has also identified an ubiquitin-binding domain (UBD) in GTPCH1, consisting of residues 95–142, that mediates the non-covalent ubiquitin binding. The data that support this notion include the following: (1) GTPCH1 sequence alignment with several previously identified UBDs (Fig. 6A) suggest that residues 95–142 of GTPCH1 comprise a potential UBD; (2) recombinant GTPCH1 NΔ2 (95–142) mediated the interaction of GTPCH1 with ubiquitin chains (Fig. 6B); and (3) importantly, we further identified a hydrophobic patch (I131, Fig. 7) in the GTPCH1 UBD, which affects GTPCH1 ubiquitination (Fig. 7B). A hydrophobic patch is a typical feature of a UBD and serves as a protein-protein interaction interface between the UBD and ubiquitin [25].

We further demonstrated that the non-covalent GTPCH1/Ub binding targets GTPCH1 for proteasomal degradation. The ubiquitin-proteasome system plays a pivotal role in protein degradation to maintain intracellular homeostasis [36]. Target proteins have different affinities for diverse lengths of ubiquitin chains [34], [35] and the length of the ubiquitin chain (particularly Ub4) is critical to signal proteolysis [27]. Different ubiquitin chains linked through different lysine residues on ubiquitin direct the marked proteins to different fates. The canonical Lys48-linked ubiquitin chain is used to signal proteolysis and the Lys63 linkage is used in several nonproteolytic signaling pathways [27]. In the present study, several lines of evidence indicate that the non-covalent interaction between polyubiquitin and GTPCH1 dictates its proteasomal degradation. First, recombinant GTPCH1 directly binds Lys48-linked Ub4 (K48-Ub4), which usually signals proteolysis [27], and specifically binds high-molecular weight ubiquitin chains (Fig. 5). Second, GTPCH1 non-covalently binds polyubiquitin, and inhibition of the 26S proteasome causes accumulation of GTPCH1/Ub complex (Fig. 4), indicating that the GTPCH1/Ub complex is subsequently degraded by the proteasome. Finally, the present study provides direct evidence that polyubiquitin targets GTPCH1 for degradation. Increasing the amount of polyubiquitin in HEK293 cells by ectopic expression of ubiquitin plasmids dose-dependently degraded the co-expressed GTPCH1 protein (Fig. 3). Furthermore, Lys48-linked, but not Lys63-linked, ubiquitin chains specifically directed GTPCH1 degradation. Indeed, increasing the amount of available ubiquitin as well as fluctuations in the intracellular ubiquitin content can actively alter the degradation of steady-state proteins [30].

GTPCH1 degradation causes a rapid decrease in BH4 [13], which are commonly observed in neurological and cardiovascular disorders [8], [10]–[17]. The adequate and continuous supply of BH4 is crucial for cellular activity [37], because BH4 is the essential cofactor for the synthesis of the neurotransmitters dopamine, norepinephrine, and serotonin [1], [2]. BH4 is also critical for the maintenance of the oxidative environment within cells through its function as a cofactor of NOS. Failure of the NOS system results in increased amounts of free radicals and, consequently, an impaired mitochondrial electron transport chain that ultimately decreases the cellular production of adenosine-5′-triphosphate [3]–[6]. BH4 deficiency has been linked to many neurological and cardiovascular disorders, including atypical phenylketonuria, dystonia, Parkinson's disease, Alzheimer's disease, depression, schizophrenia [1], [2], [5], [37], diabetes mellitus [13]–[15], [38], and hypertension [10]–[12], [39].

In summary, this is the first report of the novel non-covalent interaction between GTPCH1 and polyubiquitin, mediated by a UBD (residues 95–142). These results establish GTPCH1 as a direct target of polyubiquitin, and indicate that the UBD in GTPCH1 promotes polyubiquitin chain recognition and binding. The non-covalent interaction between polyubiquitin and GTPCH1, and the degradation of GTPCH1, provides a novel mechanism for GTPCH1 function modulation in normal physiology and human disease.
